# Inference of causality in epidemics on temporal contact networks

**DOI:** 10.1038/srep27538

**Published:** 2016-06-10

**Authors:** Alfredo Braunstein, Alessandro Ingrosso

**Affiliations:** 1 Politecnico di Torino, Corso Duca degli Abruzzi 24, 10129 Torino, Italy; 2 Collegio Carlo Alberto, Via Real Collegio 30, 10024 Moncalieri, Italy; 3 Human Genetics Foundation, Via Nizza 52, 10126 Torino, Italy

## Abstract

Investigating into the past history of an epidemic outbreak is a paramount problem in epidemiology. Based on observations about the state of individuals, on the knowledge of the network of contacts and on a mathematical model for the epidemic process, the problem consists in describing some features of the posterior distribution of unobserved past events, such as the source, potential transmissions, and undetected positive cases. Several methods have been proposed for the study of these inference problems on discrete-time, synchronous epidemic models on networks, including naive Bayes, centrality measures, accelerated Monte-Carlo approaches and Belief Propagation. However, most traced real networks consist of short-time contacts on continuous time. A possibility that has been adopted is to discretize time line into identical intervals, a method that becomes more and more precise as the length of the intervals vanishes. Unfortunately, the computational time of the inference methods increase with the number of intervals, turning a sufficiently precise inference procedure often impractical. We show here an extension of the Belief Propagation method that is able to deal with a model of continuous-time events, without resorting to time discretization. We also investigate the effect of time discretization on the quality of the inference.

Identifying past features of an epidemic outbreak remains a challenging problem even for simple stochastic epidemic models, such as the susceptible-infected (SI) model and the susceptible-infected-recovered (SIR) model. In recent years, this problem has received considerable attention, especially on discrete time models^1^,^2^,^3^,^4^,^5^. For these models, we recently proposed an approximate Bayesian method based on Belief Propagation (BP)^6^,^7^, that gave the first exact tractable solution to a family of discrete time inference problems on acyclic graphs and an excellent approximation on general graphs, including real ones. The problem addressed ranged from the inference of the epidemic source (the patient zero or index case), inference of the infection times and the epidemic parameters, all from the knowledge of the network plus a (partial, noisy) snapshot of the infection state of the system at a given instant.

In the last years, several precise spatio-temporal information about contacts between individuals in a community have been collected, representing close proximity^8^,^9^, social or sexual interactions^10^,^11^ and more. Each dataset consists of a time-stamped list of pairs of individuals. Seeking to explore characteristics of potential outbreaks, many authors studied the disease propagation over those communities employing compartment infection models such as SI and SIR. Technically, a simple way to achieve this is by computing a weighted discrete time network. This can be done by sub-dividing the time line into subintervals of length Δ (time-steps), aggregating all contacts falling in a given interval [*t*Δ,(*t* + 1)Δ] into a time-step dependent weight 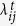 equal to 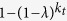, where *λ* is the probability of transmission in a single quasi-instantaneous contact and *k*_*t*_ the number of contacts the interval^8^,^6^. Once this discrete time network has been constructed, the spread of infectious diseases on the community can be described through a discrete time SIR model, in which the transition probabilities between states defining each of these models depend on the time-step *t*. However, these coarsening methods naturally lead a loss of timing information and precision, becoming exact only in the limit of small Δ and a large number of intervals. Unfortunately, the computational time of both simulations and various inference algorithms typically increase with the number of time steps, making a sufficiently precise analysis unpractical, if not impossible in most cases. In the following, we will describe a very simple semi-continuous time stochastic model of infection dynamics that does not require coarsening or binning and is naturally equivalent to the Δ → 0 limit. For simplicity, we will concentrate on the SIR model, but all methods here can be naturally generalized to other variants such as SEIR^8^. We will then develop a semi-continuous time inference framework which is able to deal with contact network datasets without any discretization approximation, may it be implicit or explicit. The method will be shown to perform very well on two datasets of real contact networks, being able to reconstruct the epidemic source, the infection times and the infection causality tree with a great degree of accuracy in a wide range of parameters. In the concluding section, its performance will be compared to a discretized version of the model, showing that the inference performance under discretization approximations, although converging to the non-discretized one, does so in a range of discretization precision that renders it extremely unpractical.

## Methods

### A static model to describe dynamics

Let us consider an evolving contact network *G* composed of *N* nodes. Each node *i* is equipped with a time dependent state variable *x*_*i*_(*t*) ∈ {*S*, *I*, *R*} so that at time *t* it can be in one of three possible states: susceptible (S), infected (I), and recovered/removed (R). We will define our dynamical model as follows: let time 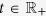 be continuous, and contagion events be instantaneous with probability *λ*. Each pair of individuals (*i*, *j*) will be in contact in a discrete set of instants *T*_*ij*_(0) < *T*_*ij*_(1) < … < *T*_*ij*_(*n*_*ij*_) (given by the real network traced dataset), where we assume for simplicity *T*_*ij*_(*r*) = ∞ for *r* > *n*_*ij*_. As time advances, contagion to node *j* will happen at a time *t*_*j*_ with probability *λ* if *i* is infected, *j* is susceptible and there exists a contact between the two nodes with some index *r*_*i*_ such that *T*_*ij*_(*r*_*i*_) = *t*_*j*_. Let us define *t*_*i*_ = min{*t*: *x*_*i*_(*t*) = *I*} the time at which node *i* gets infected (infection time) and *g*_*i*_ = min{*g*: *x*_*i*_(*t*_*i*_ + *g*) = *R*} the time passed before his recovery. Note that, as infections can only occur during contacts, each *t*_*i*_ will be necessarily equal to the time of a contact. Let us define 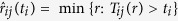 the index of the first possible such contacts. Recovery of individuals will happen at a time *t*_*i*_ + *g*_*i*_, where *g*_*i*_ follows a given recovery probability distribution with density *G*(*g*_*i*_). This parametrization is reversible, i.e. given *t*_*i*_ and *g*_*i*_, it is easy to compute the state of *x*_*i*_(*t*) at any time *t*:


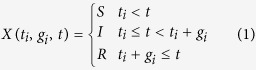


Since a node *i* has a finite probability to transmit the disease to a neighbor *j* in each of its contacts, one can compute the probability that the contagion will occur during the contact at time 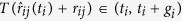, assuming that node *j* is still susceptible, simply as 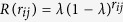. Note that after time *t*_*i*_ + *g*_*i*_, contagion will not take place because node *i* will be recovered. Given {*g*_*k*_} and {*r*_*ki*_}, infection time *t*_*i*_ of a non-source node *i* must satisfy deterministically the condition *t*_*i*_ = *F*_*i*_({*t*_*k*_}, {*g*_*k*_}, {*r*_*ki*_}) where


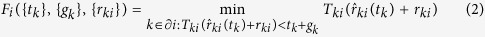


where ∂*i* denotes the set of neighbors of node *i* (i.e. nodes that share at least a contact with *i*) and it is conventionally assumed that the min is equal to +∞ if the set is empty. Eq. (2) must hold because each neighbor *k* will be in the infected state in the time interval 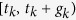, and will transmit at time 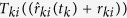; the transmission that arrives first will be the one that succeeds. Suppose now an epidemic spreading is initiated by a spreader node *i*_0_, which was infected at some time −*ε* < 0 before the first contact, which we conventionally fix at time *t* = 0. Our aim is to infer the initial spreader from just a single (possibly incomplete) observation of the state **x**(*T*) of the network at a later time *T*. The posterior distribution over the initial state of the networks can be easily written by means of Bayes theorem. In order to identify the initial spreader we should, in principle, maximize over the following posterior marginal probability:


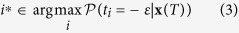


we will give a very small prior probability *γ* to each initial seed, ensuring that configurations with more than one seed are overwhelmingly improbable (note that having at least one seed is necessary to explain any evidence of infection). The prior distribution *S*(*t*_*i*_) for the node *i* then reads:


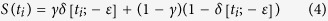


where 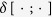 denotes the Kroenecker delta.

It is easy to see that (3) does not depend on *ε*. Indeed, for any *ε*′ > *ε*, 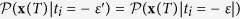

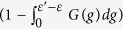
 implying that the two posteriors only differ by a constant factor, that has no relevance in (3).

The posterior distribution of infection times can now be written as 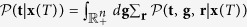 where


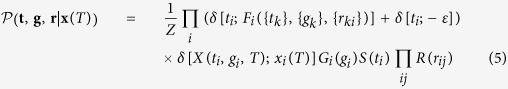


The patient zero problem can be recast as the one of computing single site marginals 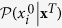 from the posterior distribution in equation (3). The problem of computing marginals over large dimensional probability distributions is in general intractable (NP-hard). In analogy with a previously introduced approximation method^6^,^7^, we will tackle this problem by means of Belief Propagation, a method which is exact on acyclic graphs, and that was shown to perform very well on random and real contact networks in the discrete time scenario.

For each node in the network, the BP algorithm provides an estimate of the posterior probability that the node got infected at a certain time, and thus also the probability that the node was the origin of the epidemics.

### Graphical model formulation

In order to apply BP, we will first formulate an alternative expression with no continuous variables, as the numerical representation of their distributions is problematic. Note that variables *t*_*i*_ are already discrete, as they live in the finite subset of the real line formed by all incoming contact times 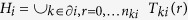. Let us consider the ordered sequence of values *T*_*i*_(0) < … < *T*_*i*_(*n*_*i*_) in this subset, and define *T*_*i*_(*n*_*i*_ + 1) = ∞. To cope with continuous variables *g*_*i*_, we will define from *g*_*i*_ a discrete variable 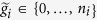, by exploiting the fact that 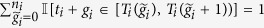 (here 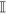 is the indicator function of the condition in its argument), and that *X* and *F* are constant for *g*_*i*_ inside an interval 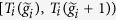. We can recast (3) as


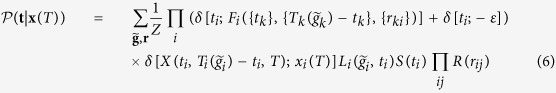


where 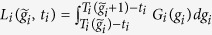. In order to simplify the structure of the probability distribution factorization, we will introduce the variables 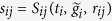 where


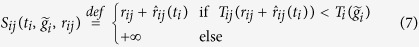


The introduction of *s*_*ji*_ variables allows to simplify the structure of equation (6):


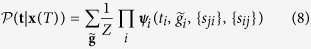


where


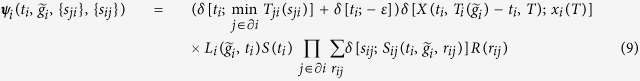


### Belief Propagation equations

To briefly describe the essence of the BP method, let us consider a probability distribution over the variables 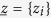 that has the following factorized form:


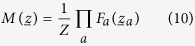


where 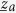 is the subset of variables that *F*_*a*_ depends on. BP equations are a set of self-consistent relations linking the so-called *cavity messages* (or *beliefs*), a set of single-site probability distributions which are associated to each directed link in the graphical model defined by the joint distribution in equation (10). The general form of BP equations is the following:


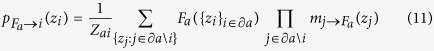



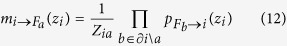



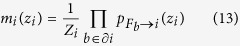


where *F*_*a*_ is a *factor*, *z*_*i*_ is a variable, ∂*a* is the subset of indices of variables in factor *F*_*a*_ and ∂*i* is the subset of factors that depend on *z*_*i*_. The terms *Z*_*ia*_, *Z*_*ai*_ and *Z*_*i*_ are local partition function, serving as normalizations. To solve equations (11) and (12) an iterative procedure is typically used, where the cavity messages are initialized with homogeneous distributions and they are asynchronously updated until convergence to a fixed point^12^,^13^. While the computation of equation (12) is straightforward, the summation in (11) often involves a number of steps growing exponentially with the size of ∂*a*. In a number of interesting contexts, though, it is possible to devise efficient methods for computing this sum, and so reducing the computational complexity of the BP updates.

The inference problem of equation (8) is interpreted as a partial marginalization of a factorized distribution as in equation (10). In this settings, there are only two types of BP message, namely 
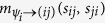
 and 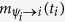, and the corresponding updates are derived straightforwardly from equation (11). The node-to-factor BP messages, namely namely 
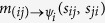
 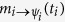, can be computed very easily by virtue of equation (12).

Calling 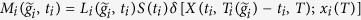, the BP equations for ***ψ***_*i*_ are


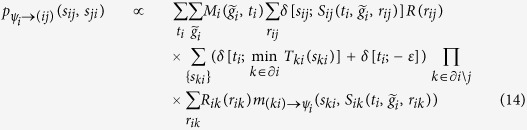



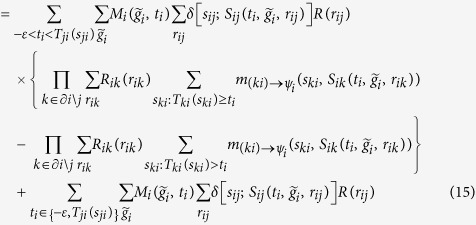



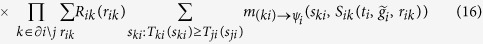


where (15) follows because


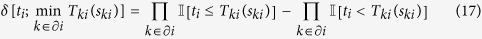



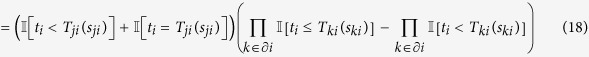



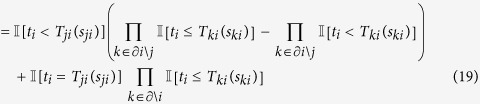


Similarly,


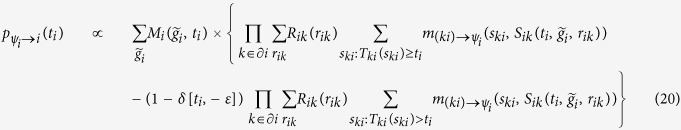


Note that values 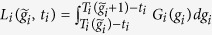 can be pre-computed in a matrix 
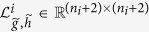
 as each *t*_*i*_ must be equal 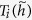 for some 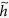 in {0, …, *n*_*i*_ + 1}. The calculation of 16, 20 can be performed more efficiently by observing that all terms


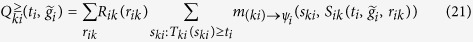



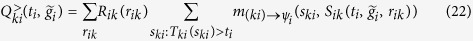


can be computed in time 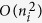. Then equations (14)–(20), 42, 43, 44, 45, 46, 47 can be computed as


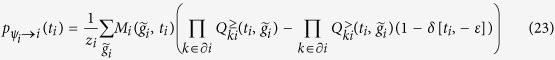



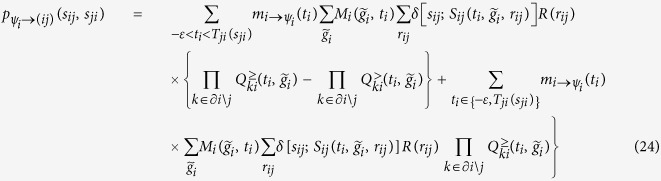


on a loop over the possible values of 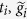.

The pseudocode in Algorithm 1 illustrates the implementation of our BP algorithm in detail. Please note that at each iteration we store factor-to-variable messages and marginals for each variable, the variable-to-factor messages being easily extracted at each iteration in view of the simple relation:


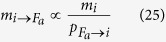



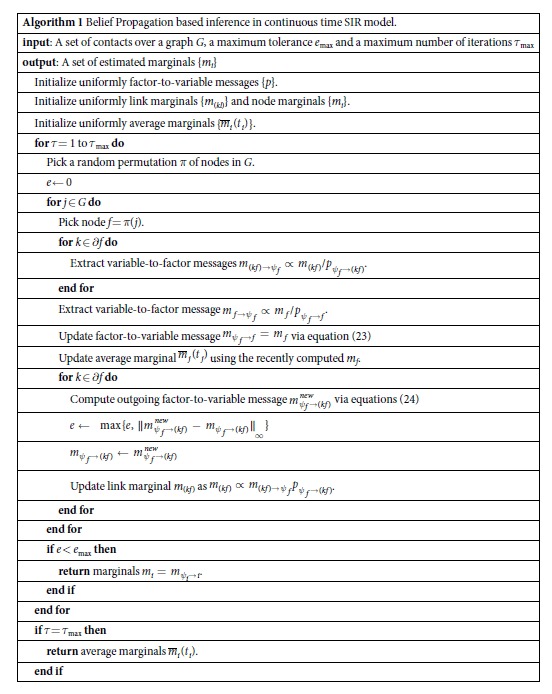


The time complexity of the computation of all messages 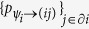 exiting node *i* is 
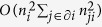
. Assuming a bounded number of contacts per individual *n*_*i*_, this scaling results in an update with a number of operations per BP iteration which is linear in the number of edges (i.e. the number of pairs of individuals that are in contact) in the full system. On a BP fixed point, equation (13) is used to compute the marginal probability *m*_*i*_(*t*_*i*_ = −*ε*), which brings an estimation of the posterior probability 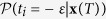 for the node to be the active before the first contact. Note that, in the present case, the marginal *m*_*i*_(*t*_*i*_) is equal to the message 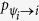. In the event that BP doesn’t converge, reliable information can be extracted equivalently from the average value over iterations of the marginals *m*_*i*_(*t*_*i*_), that we call 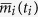.

## Results

### Patient zero detection in two large real contact time networks

We tested our methods on two large evolving networks: a database of time-stamped sexual interactions and a network of face-to-face contacts in a high school.

For each dataset, we simulated a large number of epidemic propagations, each one initiated from a unique random source (the patient zero, or seed). The nodes in the network are then ranked in decreasing order of their estimated posterior probability of being the origin of the observed epidemics: the position of the true origin in the ranking provided by the algorithm is a good measure of the efficacy of the method. In what follows, *i*_0_ stands for the index of the true origin of the epidemics, its rank being indicated by *r*_0_ = *rank*(*i*_0_). For simplicity, we will always consider homogeneous epidemic parameters *λ*_*ij*_ = *λ* and *G*(*g*_*i*_) an exponential distribution with rate constant *μ*.

The first dataset comes from a database of sexual encounters between clients and escorts on a Brazilian website, covering the beginning of the community, September 2002 through October 2008, and composed of a total of *E* = 50185 contacts between between 6642 escorts and 10106 sex-buyers. This kind of data are particularly relevant in the study of spreading of Sexually Transmitted Infections (STI), and have been previously used to model the diffusion of HIV by means of simple SI-SIR compartmental models^11^. We build a bipartite evolving network, focusing on the last two available years of operation of the website (*E* = 29628 contacts, slightly over half of the dataset) in order to skip the initial period where reporting of encounters are very sparse and incomplete. For each value of *λ*, we simulate *M* = 1000 single source epidemic propagations, with a recovery rate equal to *μ* = 0.5/*year*. Concerning algorithmic efficiency, the efficient implementation of the BP updates presented in the previous section allows us to perform inference in large-scale contact network with a remarkably small computational cost. As an example, a parallel C++ implementation with 5 concurrent processes took roughly 11 minutes to converge to a solution with *rank*(*i*_0_) = 1 (perfect identification of the source) on a single instance with an epidemic size of *N*_*IR*_ = 1810 individuals, obtained with *λ* = 0.4 and *μ* = 0.5/*year*.

**Figure 1:**
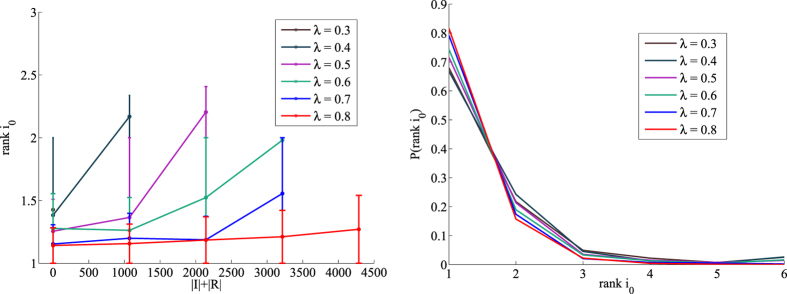
Left: Average absolute rank *r*_0_ of the true patient zero as a function of the epidemic size *N*_*IR*_ = |*I*| + |*R*| for *μ* = 0.5/*year* and increasing values of the infection probability *λ* in the network of sexual contacts. Each curve represents a sample of *M* = 1000 random instances. Lines are guide to the eye. Right: probability distribution of *rank i*_0_ over the same epidemics for each value of *λ*.

In Fig. 1 we show the average absolute rank *r*_0_ of the true first infected individual *i*_0_ as a function of the epidemic size *N*_*IR*_ = |*I*| + |*R*| (i.e. the number of infected and recovered sites), whose values are discretized in intervals of width equal to 0.1. The low values of the rank show the effectiveness of the method.

The second dataset consists of a collection of Close Proximity Interactions (CPIs) obtained by means of wireless sensor network technology (TelosB motes)^8^. Data were collected in a US high school and provide an almost complete account of face-to-face interactions during a whole day at school. All in all 798 individuals were monitored, corresponding to the 94% of the total school community, and 2148991 unique Close Proximity Records (CPR) were acquired. A single CPR corresponds to a close proximity detection event between two motes (max. 3 meters). The authors of the study perform an aggregation of the raw data in *interactions*, defined as continuous sequences of CPRs between the same two nodes. Our choice was to go back to the raw data and investigate the spreading process at the level of single CPRs, using the intensity signal as a proxy for the closeness of a face-to-face contact (a detailed account is present in Salathe *et al* - Supplementary Information^8^). We constructed a set of evolving networks by setting a threshold *θ*_*int*_ for the signal intensity of the motes, thus resulting in denser networks for smaller *θ*_*int*_, where more weaker (and distant) contacts are taken into account. A second interesting possibility could be to allow for a probability of contagion that depends on the proximity (i.e. the strength of the signal). We did not persue this route as the dependence of probability cf contagion on the distance is hard to determine (to our knowledge, no study provides this information for known diseases), and moreover we don’t have information on the correspondence between signal strength and distance. Besides, a threshold-like dependence on the distance could be adequate for contagion of many infectious diseases, such as non-airborne ones. In any case, the analysis technique presented here can be adapted directly to the case of contact-dependent probability would the needed modelling information mentioned above be available in the future.

**Figure 2:**
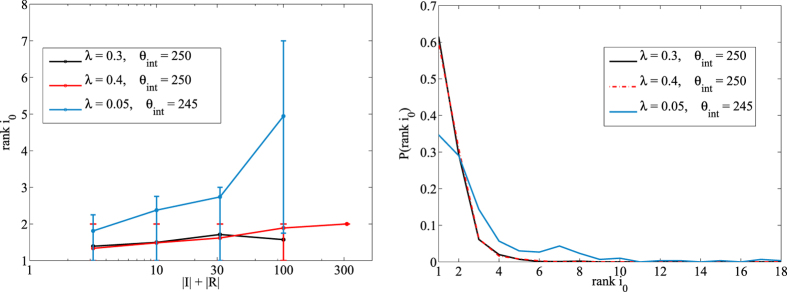
Left: Average abolute *r*_0_ = *rank*(*i*_0_) as a function of the total epidemic size *N*_*IR*_ = |*I*| + |*R*| in the network of face-to-face contacts in a high school for different values of the threshold *θ*_*int*_. Each curve is an average over *M* = 1000 (*θ*_*int*_ = 250) or *M* = 300 (*θ*_*int*_ = 245) random instances. Lines are guide to the eye. Right: probability distribution of *ranki*_0_ over same epidemics for the three set of parameters.

Three representative examples are show in Fig. 2, which displays the average rank of the true first infected individual *i*_0_ for different values of *λ* and threshold *θ*_*int*_. Two values of *λ* = 0.3, 0.4 are explored for *θ*_*int*_ = 250. Then we attempted with the much denser graph resulting of considering *θ*_*int*_ = 245. Here the infection probability *λ* has been chosen to maintain the same average number of infections as the case *θ*_*int*_ = 250, *λ* = 0.3.

### Reconstruction of causality

Consider the problem of inferring for each non-susceptible individual *i* at time *T*, the individual *k* from which he contracted the infection. The probability *p*_*ktoi*_ of such transmission event corresponds to


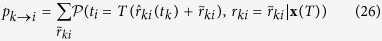


**Figure 3:**
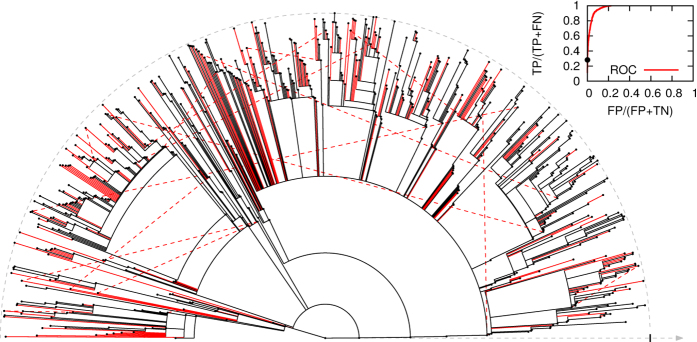
Reconstruction of the causal history of transmissions on a simulated epidemic outbreak on a sexual contact network infecting 719 individuals (black dots) from a snapshot of the infection state at time 2y (corresponding to the outer dashed semicircle). The figure represents the tree of transmissions in the outbreak originated in the central node (time flows in the outwards radial direction). Radial segments correspond to 718 true transmission events. The 4130 oriented pairs of infected individuals that were in contact were ordered by their decreasing estimated posterior probability of transmission. A ROC curve (area equal to 0.898) of the ordered list is shown in the top right panel, with the black point corresponding to the main figure. In the main figure, red full radial segments correspond to 202 correctly inferred transmissions (true positives, TP), black ones to non-inferred transmissions (false negatives, FN) and red dashed lines to the 16 wrongly predicted transmissions (false positives, FP).

Once *p*_*k*→*i*_ has been computed for every (ordered) pair (*ki*), a prediction will be formed by the subset of pairs with probability larger than a given threshold. A *receiver operating charasteristic* (ROC) curve can be computed by considering the performance for all possible thresholds. The ROC curve for an instance of an outbreak on the sexual contacts dataset is shown in Fig. 3, along with the inferred pairs in one single point of the curve. It is evident that a large fraction of the entire history of the propagation can be reconstructed with a high degree of reliability, despite the apparently limited amount of information available in a single observation of the nodes’ states.

**Figure 4:**
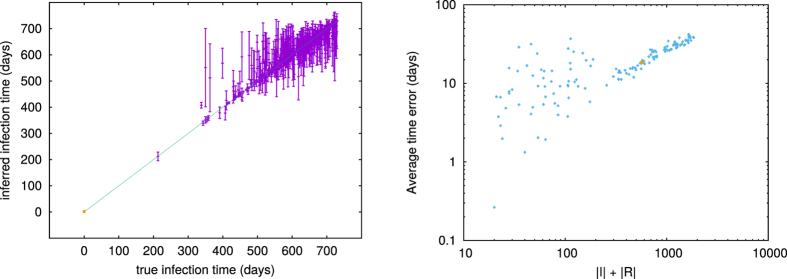
Left panel: inferred vs true infection time for a single epidemic cascade in the network of sexual contacts. Epidemic parameters are *λ* = 0.4 and *μ* = 0.5/*year*. The vertical bars represent the standard deviation of the inferred infection time computed from the BP marginals. The zero patient, highlighted in orange, is correctly identified as the first infected individual. Right panel: Average Time Error (*ATE*) sorted as a function of the epidemic size *N*_*IR*_ in 140 samples (we simulated 200 epidemic cascades and then focused on samples with *N*_*IR*_ > 20, where enough information is present at the time of observation). Epidemic parameters are the same as in the left plot, each point represents the *ATE* value for a single epidemic cascade. The orange dot corresponds to the cascade on the left panel.

For a comparison between the true infection times 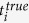 of each node and the ones that can be inferred by our method, we show in the left panel of Fig. 4 a scatter plot of 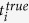 versus 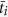 for a single epidemic cascade in the network of sexual contacts, with epidemic parameters *λ* = 0.4 and *μ* = 0.5/*year* (in the resulting epidemics the number of infected individuals is |*I*| = 991, total epidemic size being *N*_*IR*_ = 1070). The infection times 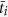 have been simply obtained by averaging over the marginal posterior distribution 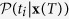 of single-node infection times. In addition, we show in the right panel of Fig. 4 the Average Time Error (*ATE*) between **t**^*true*^ and 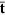, which we define as 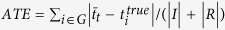, for a set of 200 different samples of simulated epidemic outbreaks in the same network and with the same epidemic parameters as in the previous example. The reconstruction of the dynamical history is remarkably good over a wide range of epidemic sizes.

### Partial and noisy observations

It is not difficult to extend the present model to account for observations affected by some kind of uncertainty. One simply introduces the Observational Transition Matrix (OTM) *o*_*i* _(*y*_*i*_|*x*_*i*_), containing the transition probabilities from the true state *x*_*i*_ to the observed state: in order to perform inference, one has to some over all the possible true unobserved states with a weight given by the corresponding entry in the OTM, a task which is easily accomplished in the BP inference scheme. The identity matrix *o*_*i*_(*y*_*i*_|*x*_*i*_) = *δ*[*y*_*i*_; *x*_*i*_] corresponds to the a case in which no noise enters the observations. Please note that, in this generalized scheme, we can simply take into account partial observability with a totally flat OTM 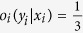 for unobserved nodes.

Firstly, we consider the case of partial observations, i.e. the case in which only a subset of nodes are accessible for observation at time *T*: this is the standard realistic scenario in practical applications, when a complete monitoring of a full network is infeasible in the general case. We simulate a number of epidemic spreading in the contact network and model the partial observability by a fixed probability *p*_*ob*_ of observing a node at time *T*. In what follows, we will use the sexual encounters dataset, mostly because of its epidemiological relevance.

**Figure 5:**
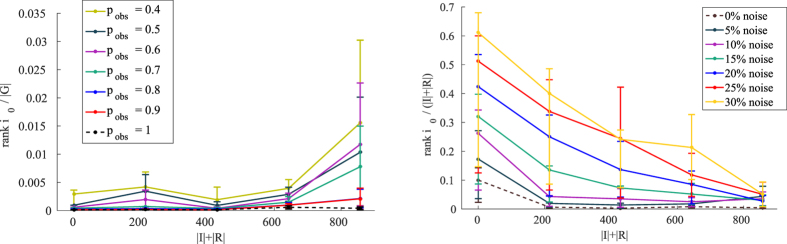
Inference performance with partial or noisy observations in the network of sexual contacts. In each curve we show the average normalized rank over *M* = 100 random instances of the true patient zero *i*_0_ as a function of the total epidemic size *N*_*IR*_ = |*I*| + |*R*| for *λ* = 0.2, *μ* = 0.5/*year*. Left panel: *r*_0_ = *rank*(*i*_0_) normalized over |*G*| vs *N*_*IR*_ for different values of probability of observation *p*_*obs*_; dashed curve is the case with full observations. Right panel: normalized *r*_0_ = *rank*(*i*_0_) vs *N*_*IR*_ for different noise intensity *ν*; dashed curve is the case with no noise. Lines are guide to the eye.

Results for decreasing values of *p*_*ob*_ in the network of sexual contacts are shown in the left panel of Fig. 5 (the complete observation case *p*_*ob*_ = 1 is shown in the dashed line for reference). The BP method happens to be highly resilient even with a high amount of hidden information at time of observation *T*.

Turning to the problem of uncertain observations, we use a very simple symmetric model for observational noise. Let us consider the following OTM:


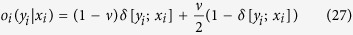


This matrix describes a kind of symmetric noise, where a node that is in the state *x* has a probability 1 − *ν* of being correctly observed in its state, and a probability *ν* of being observed incorrectly in one of the other two states. Suppose, for example, node *i* is S (susceptible) at observation time *T*: for a given noise probability *ν*, there is an equal probability 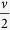 for the node *i* to be observed in the *R* (recovered) or *I* (infected) state - the same holds for the others two cases, equivalently. In Fig. 5, right panel, we show that our BP algorithm is highly robust even to a significant amount of noise, up to 30%.

### Discretization and binning

In order to ascertain the eventual loss of inference precision due to time-discretization, we performed the following experiment on the sexual intercourse dataset^10^. We generated *M* = 100 random epidemics with the semi-continuous time model with instantaneous probability of transmission *λ*.

Separately, for each value of *T* = 10, 15, 20, 25 we produced a discrete time temporal network from by sub-dividing the time interval [*t*_0_, *t*_1_] into *T* equal subintervals (time-steps) of length Δ = (*t*_1_ − *t*_0_)/*T*, aggregating all contacts falling in a given interval [*t*Δ; (*t* + 1)Δ] into a time-step dependent weight 
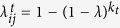
, where *k*_*t*_ is the number of contacts in the interval^8^,^6^.

**Figure 6:**
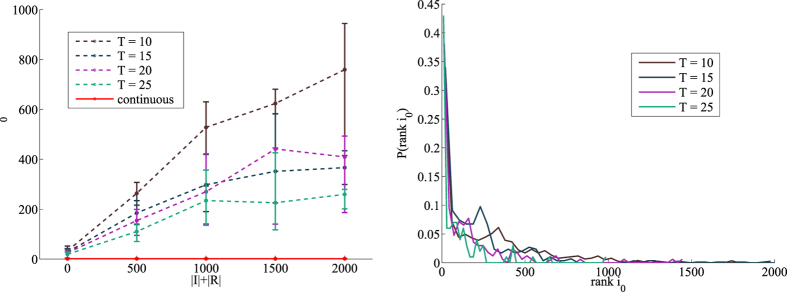
Left: Comparison between the continuous time method and the discretized version in the network of sexual contacts for infection probability *λ* = 0.4 and *μ* = 0.5/*year*. Each curve is the average absolute rank *r*_0_ over *M* = 100 samples as a function of the epidemic size *N*_*IR*_. Full curve: continuous time version. Dashed curves: contacts have been aggregated in effective contacts so that final time of observation *T* is systematically increased from *T* = 10 to *T* = 25. Lines are guide to the eye. Right: probability distribution of *rank i*_0_ over the same epidemics for the four values of *T*. The distribution for the continuous case has been omitted since probability is extremely concentrated around *r*_0_ = 1 and appears off-scale.

We then performed the inference analysis on the *M* previously generated epidemics both in the discretized network with the the discrete-time BP algorithm^6^ and using the semi-continuous time inference BP algorithm presented here. We investigated the difference in performance for different combinations of the epidemic parameters, noting that the semi-continuous time method strikingly outperforms the discretization procedure in all cases. As an example, we show in Fig. 6 a comparison of the continuous time method versus the discretized version for *λ* = 0.4 and *μ* = 0.5/*year* with an increasing number of time bins.

## Discussion

In this work, we developed an inference framework to analyze the dynamics of infection in temporal contact networks with continuous or very fine-grained time resolution. We showed by means of simulations on real contact networks how the approach is able to reconstruct with great degree of accuracy both the source of the epidemics, the infection times and the underlying epidemic causal history from the mere observation of the state of the system (noisy or incomplete) at a single instant in a wide range of parameters.

Moreover, we were able to quantify the loss of information due to time-discretization, demonstrating a remarkable improvement with continuous time inference when compared with time discretized data even for a relatively large number of time sub-intervals.

It would be interesting to apply this technique on other relatively closed communities where the interactions can be monitored but the infections themselves are hidden, such as in hospital wards^14^ and for applications to computer virus forensics^4^. The ability to reconstruct the epidemic history and causality of transmissions could prove to be helpful to devise better containment strategies. In those cases, generalizations of the method to epidemic models related to SIR such as SEIR and other distributions of recovery time different from exponential could be necessary.

## Additional Information

**How to cite this article**: Braunstein, A. and Ingrosso, A. Inference of causality in epidemics on temporal contact networks. *Sci. Rep.* **6**, 27538; doi: 10.1038/srep27538 (2016).

## References

[b1] Antulov-Fantulin N., Lancic A., Stefancic H., Sikic M. & Smuc T. Statistical Inference Framework for Source Detection of Contagion Processes on Arbitrary Network Structures. In *2014 IEEE Eighth International Conference on Self-Adaptive and Self-Organizing Systems Workshops (SASOW)*, 78–83 (2014).

[b2] Lokhov A. Y., Mézard M., Ohta H. & Zdeborová L. Inferring the origin of an epidemic with a dynamic message-passing algorithm. Phys. Rev. E 90, 012801 (2014). 10.1103/PhysRevE.90.012801 25122336

[b3] Pinto P. C., Thiran P. & Vetterli M. Locating the source of diffusion in large-scale networks. Physical Review Letters 109, 068702 (2012). 23006310 10.1103/PhysRevLett.109.068702

[b4] Shah D. & Zaman T. Detecting sources of computer viruses in networks: theory and experiment. ACM SIGMETRICS Performance Evaluation Review 38, 203–214 (2010).

[b5] Shah D. & Zaman T. Rumors in a network: Who’s the culprit? Information Theory, IEEE Transactions on 57, 5163–5181 (2011).

[b6] Altarelli F., Braunstein A., Dall’Asta L., Lage-Castellanos A. & Zecchina R. Bayesian inference of epidemics on networks via belief propagation. Physical Review Letters 112, 118701 (2014). 24702425 10.1103/PhysRevLett.112.118701

[b7] Altarelli F., Braunstein A., Dall’Asta L., Ingrosso A. & Zecchina R. The patient-zero problem with noisy observations. Journal of Statistical Mechanics: Theory and Experiment 2014, P10016 (2014).

[b8] Salathé M. A high-resolution human contact network for infectious disease transmission. Proceedings of the National Academy of Sciences 107, 22020–22025 (2010). 10.1073/pnas.1009094108 PMC3009790 21149721

[b9] Isella L. What’s in a crowd? analysis of face-to-face behavioral networks. Journal of Theoretical Biology 271, 166–180 (2011). 21130777 10.1016/j.jtbi.2010.11.033

[b10] Rocha L. E. C., Liljeros F. & Holme P. Information dynamics shape the sexual networks of internet-mediated prostitution. Proceedings of the National Academy of Sciences 107, 5706–5711 (2010). 10.1073/pnas.0914080107 PMC2851932 20231480

[b11] Rocha L. E. C., Liljeros F. & Holme P. Simulated epidemics in an empirical spatiotemporal network of 50,185 sexual contacts. PLoS Comput Biol 7, e1001109 (2011). 21445228 10.1371/journal.pcbi.1001109 PMC3060161

[b12] Yedidia J. S., Freeman W. T. & Weiss Y. Bethe free energy, kikuchi approximations, and belief propagation algorithms. Advances in neural information processing systems 13 (2001).

[b13] Mézard M. & Montanari A. Information, Physics, and Computation (Oxford University Press, 2009).

[b14] Vanhems P. Estimating Potential Infection Transmission Routes in Hospital Wards Using Wearable Proximity Sensors. PLoS ONE 8, e73970 (2013). 24040129 10.1371/journal.pone.0073970 PMC3770639

